# Targeting serine/glycine metabolism to attenuate IFN-γ- and IL-22-driven inflammation and hyperproliferation in psoriasis

**DOI:** 10.1038/s41420-026-03138-3

**Published:** 2026-05-08

**Authors:** Laura Mercurio, Valentina Di Francesco, Simone Sergio, Martina Morelli, Claudia Scarponi, Stefania Madonna, Sabatino Pallotta, Mara Mancini, Angela Cappello, Eleonora Candi, Cristina Albanesi

**Affiliations:** 1 Experimental Immunology Laboratory and Department of Dermatology, IDI-IRCCS, 00167 Rome, Italy; 2 Biochemistry Laboratory, IDI-IRCCS, 00167 Rome, Italy; 3 Dept. of Experimental medicine, Tor Vergata University of Rome, 00133 Rome, Italy

**Keywords:** Mechanisms of disease, Cytokines

## Abstract

Psoriasis is a chronic skin disease characterized by keratinocyte hyperproliferation and inflammation, largely driven by the cytokines IL-22 and interferon (IFN)-γ. These cytokines activate the signal transducer and activator of transcription (STAT) 3 and STAT1 molecular pathways, leading to abnormal proliferation, impaired differentiation, and increased production of inflammatory mediators in keratinocytes. While the IL-22/STAT3 pathway primarily promotes de-differentiation in keratinocytes, IFN-γ/STAT1-3 signaling induces pronounced inflammation, despite exerting antiproliferative effects on these cells. Recent research has highlighted the role of serine/glycine metabolism in the pathogenesis of psoriasis, by supporting T cell and keratinocyte proliferation. Furthermore, pharmacological inhibition of serine catabolism through targeting serine hydroxymethyltranferase (SHMT)1/2 enzymes reduced the infiltration of inflammatory cells in the skin of the imiquimod-induced mouse model of psoriasis. This study investigates the role of serine catabolism in psoriasis, focusing on its influence on keratinocyte proliferation and inflammation. We examined how pharmacological inhibition of SHMT1/2, mediated by a folate-competitive cell-permeable inhibitor Serine Hydroxymethyltransferase INhibitor 1 (SHIN1), affects keratinocyte proliferation and inflammatory signaling pathways in response to psoriasis-associated cytokines IL-22 and IFN-γ, using both in vitro and ex vivo models of the disease. We found that SHIN1 reduced keratinocyte proliferation, particularly under IL-22 stimulation, and restored differentiation in ex vivo psoriasis skin explants by reversing the effects of IL-22. SHIN1 also inhibited IFN-γ-induced expression of pro-inflammatory genes (e.g., *CXCL10*, *CXCL9*, *CCL5*, *CCL2*, *IL-6*) and reduced STAT3 activation, with only modest effects on STAT1 and extracellular signal-regulated kinase 1/2 activation. In psoriasis explants, SHIN1 decreased the expression of Ki67, Keratin 16, and pro-inflammatory cytokines including IL-17A, IL-22, and IFN-γ. These findings support the therapeutic potential of SHIN1 as a metabolism-targeted agent for psoriasis and other cytokine-mediated skin disorders, providing a rationale for further exploration of novel treatment strategies.

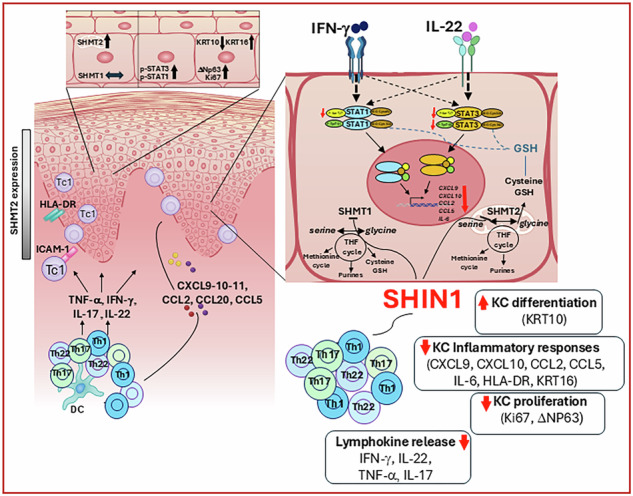

## Introduction

Psoriasis is a chronic immune-mediated inflammatory skin disorder characterized by abnormal keratinocyte proliferation and differentiation driven by sustained immune activation and persistent inflammation [1, 2]. Immune responses are initiated by both endogenous and exogenous danger signals and amplified by cytokines that activate innate pathways followed by T cell-mediated adaptive immunity [3, 4]. Dendritic cells (DCs) play a central role in disease pathogenesis by presenting antimicrobial peptides (AMPs) and other overexpressed self-antigens complexed with nucleic acids from damaged keratinocytes [3]. In addition, DCs shape pathogenic T-cell responses through the production of TNF-α, IL-23, and IL-12, which promote T helper (Th)17 and Th1 differentiation. These effector T cells establish a cytokine milieu dominated by IL-17, IL-22, IFN-γ, and TNF-α, thereby driving keratinocyte proliferation, impairing terminal differentiation, and sustaining chronic inflammation [5, 6]. In this highly dynamic inflammatory microenvironment, metabolic reprogramming is increasingly recognized as a critical determinant of keratinocyte growth and inflammatory activation [7].

Recent work has emphasized the importance of cellular metabolism in psoriasis, particularly in keratinocytes, which require large amounts of energy and biosynthetic precursors to sustain accelerated proliferation [8, 9]. Among these metabolic pathways, serine metabolism has emerged as a key regulator of anabolic growth. Serine is a nonessential amino acid that can be acquired from diet or synthesized de novo from glucose through the serine biosynthetic pathway [10]. Beyond its role as a proteinogenic amino acid, serine contributes to one-carbon metabolism, nucleotide biosynthesis, redox homeostasis, and cellular stress responses [11, 12].

Serine is metabolized by SHMT enzymes, which catalyzes the conversion of serine and tetrahydrofolate into glycine and 5,10-methylenetetrahydrofolate. This enzyme plays a central role in maintaining cellular homeostasis and supporting nucleotide and lipid biosynthesis, and NADPH production [13–15].

Extracellular serine is therefore essential for anabolic processes in proliferating cells [13, 16, 17], particularly in cancer, where deregulated SHMT expression sustains altered one-carbon metabolism and tumor growth [18–20]. In non-melanoma skin cancer (NMSC), for example, SHMT2 and MTHFD2 are upregulated in basal cell and squamous cell carcinomas, and silencing these enzymes disrupts proliferation in epidermoid cancer cultures [21].

Serine metabolism is also critical for immune cell and keratinocyte proliferation [15, 17]. Serine availability supports T-cell proliferation after antigenic stimulation [17], and dietary serine restriction limits antigen-specific T cell expansion. In previous work, we demonstrated a relevant role for serine/glycine metabolism in keratinocyte proliferation in psoriasis, where SHMT2 is markedly upregulated [15]. Pharmacological inhibition of SHMT1/2 reduced hyperproliferation of epidermal keratinocytes, limited inflammatory cell infiltration, and decreased the expression of psoriasis-associated cytokines and chemokines [15]. These findings indicate that SHMT-dependent serine metabolism contributes not only to the metabolic demands of proliferating keratinocytes but also to the amplification of local inflammatory responses. However, the mechanisms linking serine catabolism to cytokine-driven keratinocyte hyperproliferation and inflammation remain unclear.

In the present study, we investigated the contribution of serine catabolism to psoriasis pathogenesis, focusing on its role in keratinocyte proliferation and inflammation driven by psoriasis-associated cytokines. Specifically, we examined how inhibition of SHMT1/2 using the folate-competitive inhibitor SHIN1 [22] modulates inflammatory signaling pathways and proliferation in response to IL-22 and IFN-γ in both in vitro and in ex vivo models of psoriasis. By defining the metabolic control of cytokine-driven epidermal pathology, these studies provide mechanistic insights and support the development of metabolism-targeted therapeutic strategies for inflammatory skin diseases.

## Results

### SHIN1 reduces proliferation in keratinocytes modulated by psoriatic cytokines and prevents them from completing their differentiation programs

**Fig. 1:**
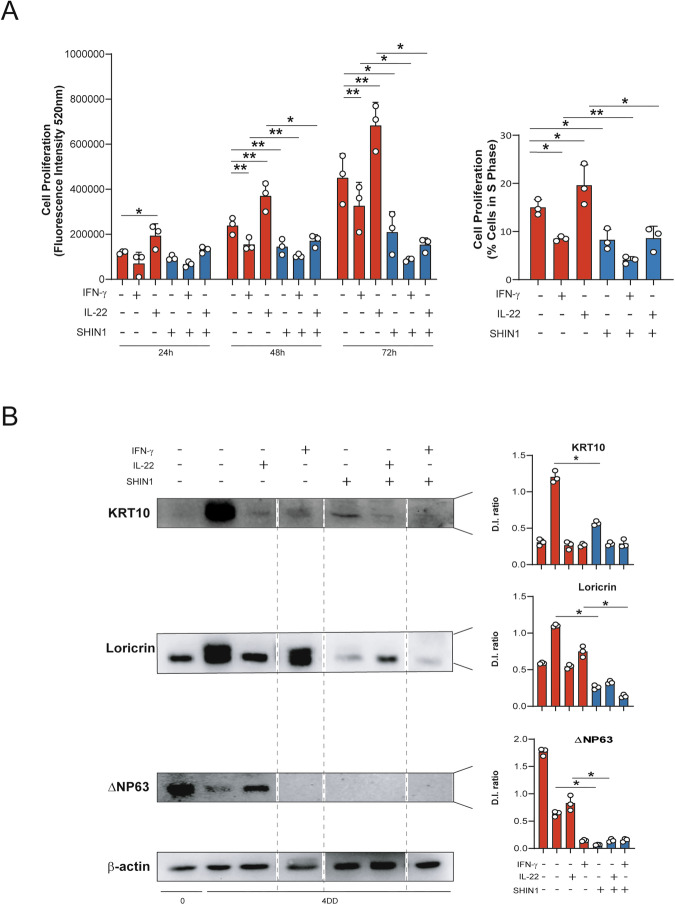
Inhibition of SHMT1/2 by SHIN1 reduces proliferation in keratinocytes modulated by IL-22 and IFN-γ and prevents them from completing their differentiation programs. **A** In the left panel, CyQUANT proliferation assay was performed to determine the growth of human keratinocyte cultures exposed to IFN-γ or IL-22, with or without SHIN1 for 24, 48, and 72 h. Data are shown as mean values of fluorescence intensity obtained from three independent experiments ± SD. In the right panel, Edu assay was conducted to evaluate cell proliferation of HEKn treated for 24 h under the same experimental conditions. Data are presented as the percentage of cells in the S phase of the cell cycle and as means ± SD (*n* = 3 independent experiments). *p < 0.05, **p < 0.01. **B** KRT10, loricrin and ΔNp63 were analysed by Western Blotting performed on keratinocyte culture pre-treated with SHIN1 and subsequently stimulated with IFN-γ and IL-22 until 4 days of differentiation. Bands corresponding to KRT10, loricrin, ΔNp63, and β-actin were cropped from the same gels/blots, with cropping points indicated by dotted lines. Graphs show densitometric intensity (D. I.) mean values obtained from three independent experiments ± SD and expressed as the ratio of protein expression levels normalized to β-actin. *p < 0.05.

The primary objective of this study was to explore the therapeutic potential of SHIN1 in psoriasis by inhibiting keratinocyte proliferation through blockade of the SHMT1 and SHMT2 enzymes, which enhance serine/glycine metabolism and support growth [15]. We first assessed keratinocyte proliferation under basal conditions and after stimulation with psoriasis-associated cytokines could be modulated by the selective SHMT1/SHMT2 inhibitor SHIN1. To this end, keratinocytes were cultured for 24, 48, and 72 h in the presence or absence of SHIN1 and treated with IL-22 or IFN-γ, which exert opposite effects on proliferation, acting as pro-proliferative and cytostatic cytokines, respectively [23–25]. IL-22 increased keratinocyte proliferation, whereas IFN-γ markedly reduced it, with a prominent effect at 48 and 72 h (Fig. 1A, left panel). CyQuant analysis showed that SHIN1 significantly reduced keratinocyte proliferation under basal conditions and after cytokine stimulation. This inhibitory effect was most evident at 48 and 72 h and was particularly pronounced in IL-22-treated cultures. SHIN1 also further potentiated the inhibitory effect of IFN-γ on keratinocyte proliferation (Fig. 1A, left panel). Consistently, SHIN1 reduced S-phase entry in keratinocytes under basal conditions or after IL-22 treatment, decreasing the proportion of S-phase cells (Fig. 1A, right panel). S-phase progression was also diminished after IFN-γ treatment, and was further reduced in the presence of SHIN1.

We next evaluated whether SHIN1 affected terminal differentiation in keratinocytes, particularly under IL-22, which promotes excessive proliferation and impairs differentiation in psoriatic skin [23]. In particular, we analyzed the expression of proliferation and terminal differentiation markers, namely, the ΔN isoform of p63 (ΔNp63) and keratin (KRT)10 and loricrin, respectively, in keratinocytes cultured for four days post-confluence following IL-22 or IFN-γ treatment, with or without SHIN1. As expected, IL-22 suppressed KRT10 and loricrin expression while increasing levels of the proliferation marker ΔN isoform of p63 (ΔNp63) (Fig. 1B). In contrast, IFN-γ reduced ΔNp63 expression and induced cell-cycle arrest in keratinocytes, preventing the completion of differentiation programs and the expression of KRT10 and loricrin (Fig. 1B). Consistent with its anti-proliferative effect, SHIN1 further reduced ΔNp63, KRT10, and loricrin in terminally differentiated keratinocytes (Fig. 1B). The effect of SHIN1 was also evident in IL-22-treated keratinocytes, as it prevented ΔNp63 upregulation and induced an additional reduction of KRT10 and loricrin expression (Fig. 1B). Full-length blots corresponding to Fig. 1B are provided in the Supplementary files.

Together, these data demonstrate that SHMT1/2 inhibition by SHIN1 reduces keratinocyte proliferation in response to psoriatic cytokines and prevents the completion of terminal differentiation programs.

### SHIN1 modulates inflammatory gene expression in experimental in vitro and ex vivo models of psoriasis

**Fig. 2:**
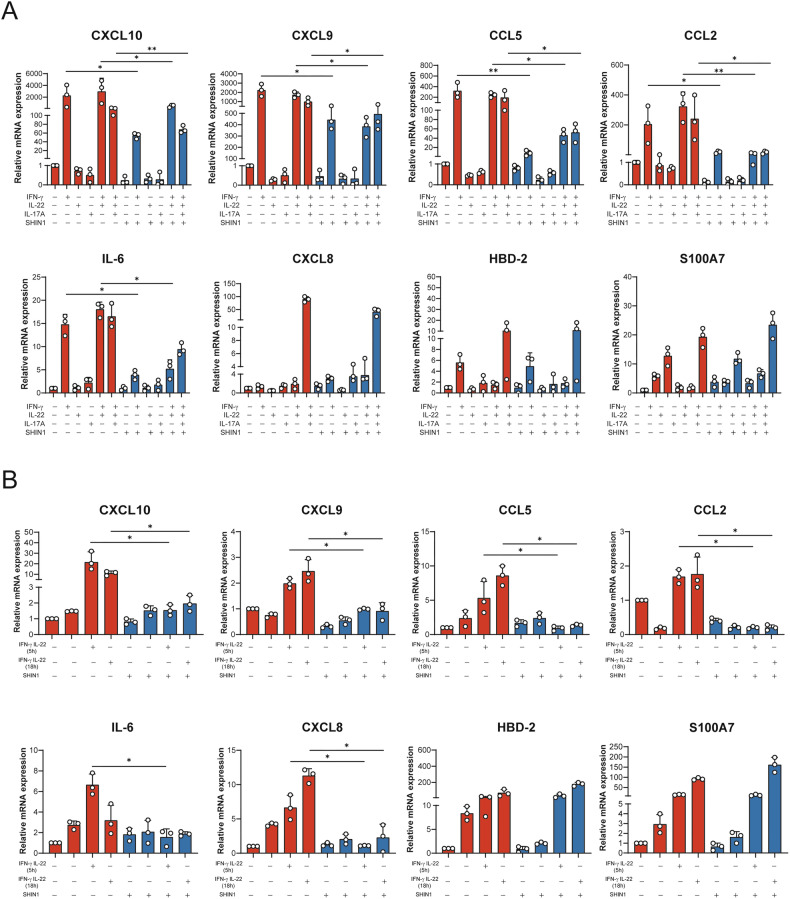
SHIN1 modulates inflammatory gene expression in cytokine-stimulated keratinocyte cultures and in ex vivo skin grafts. **A** mRNA expression levels of CXCL10, CXCL9, CCL5, CCL2, IL-6, CXCL8, HBD-2, and S100A7 were measured by RT-qPCR. Keratinocytes were pre-treated or not with SHIN1 and then stimulated with IFN-γ, IL-22 and IL-17A in different combinations for 18 h. Data were normalized to 18S rRNA levels and presented as mean ± SD (*n* = 3 independent experiments). *p < 0.05 and **p < 0.01. **B** mRNA expression of CXCL10, CXCL9, CCL5, CCL2, IL-6, CXCL8, HBD-2 and was analyzed in ex vivo skin grafts obtained from healthy donors (*n* = 3) using RT-qPCR. Human skin explants were left untreated or stimulated with IFN-γ and IL-22 cytokines for 5 and 18 h, in presence or not of SHIN1. Data were normalized to HPRT1 mRNA levels and reported as 2^-ΔCT ± SD compared to untreated controls. Values were obtained from triplicate experiments. *p < 0.05 and **p < 0.01.

In the next series of experiments, we investigated whether the inhibition of serine-dependent metabolism by SHIN1 affected the expression of inflammatory mediators in keratinocytes induced by psoriasis-related cytokines. We therefore examined the expression of key inflammatory mediators in human cultured keratinocytes, pre-treated with SHIN1 and stimulated with IFN-γ, IL-22, and IL-17A, alone or in combination. SHIN1 significantly reduced IFN-γ-reduced mRNA expression of CXCL10, CXCL9, CCL5, CCL2, and IL-6, which were strongly upregulated in keratinocytes by IFN-γ and only modestly induced by IL-22 and IL-17, even when combined with IFN-γ (Fig. 2A). In contrast, SHIN1 did not significantly affect CXCL8, human beta-defensin (HBD)-2, and S100A7 mRNA, which were preferentially induced by the three cytokines together (Fig. 2A). In parallel, flow cytometry analysis revealed that SHIN1 significantly inhibited the expression of HLA-DR, an MHC class II molecule induced by IFN-γ in keratinocytes (Table 1). Conversely, SHIN1 increased the expression of intercellular adhesion molecule (ICAM-1), possibly as a consequence of reduced cell-cell contacts resulting from proliferation inhibition. SHIN1 did not affect basal or IFN-γ-induced MHC class I expression (Table 1). These findings suggest that SHIN1 selectively inhibits specific pro-inflammatory pathways, particularly those driven by IFN-γ in cultured keratinocytes.

**Table 1:** SHIN1 inhibits membrane HLA-DR expression and upregulates ICAM1 induced by proinflammatory cytokines in cultured keratinocytes.

	HLA-DR	ICAM-I	MHC-I
Untreated	0.7 ± 0.1	1.9 ± 0.6	1.2 ± 0.3
IFN-γ	4 ± 1.4	18.6 ± 6.8	5.2 ± 1.1
IFN-γ IL-22	6.2 ± 1.2	39.7 ± 18.3	5.2 ± 2.2
IFN-γ IL-22 IL-17	6.2 ± 3.5	44.1 ± 21.7	6.3 ± 1.1
**Treatment with SHIN1:**			
SHIN1	0.9 ± 0.4	2.4 ± 0.2	1.7 ± 1.3
IFN-γ + SHIN1	1.5 ± 0.7	35.2 ± 12.1*	4.9 ± 1.0
IFN-γ IL-22 + SHIN1	1.9 ± 0.6*	77.6 ± 46.4*	5.6 ± 0.4
IFN-γ IL22 IL-17 + SHIN1	2.2 ± 1.0*	78.5 ± 45.1*	5.2 ± 1.9

*p < 0.05, cytokine-treated vs. keratinocytes co-treated with SHIN1 and cytokines.

Mean ± SD of mean fluorescence intensity of HLA-DR, ICAM-1, and MHC-I molecules on the surface of keratinocytes, assessed by flow cytometry.

To further assess the anti-inflammatory potential of SHIN1, ex vivo experiments were performed using healthy human skin explants stimulated with a cytokine mixture of IFN-γ and IL-22 to mimic in vivo psoriatic inflammation, in the presence or absence of SHIN1. Cytokine treatment markedly increased the expression of CXCL10, CXCL9, CCL2, CCL5, CXCL8, IL-6, HBD-2 and S100A7 within 5 h (Fig. 2B). Among these mediators, CXCL10, CXCL9, CCL5, CCL2, IL-6 and CXCL8 were significantly reduced by SHIN1 at both 5 and 18 h, whereas HBD-2 and S100A7 expression remained unaffected (Fig. 2B).

These results confirm that SHIN1 selectively suppress pro-inflammatory gene expression induced by IL-22 and IFN-γ in human skin.

### SHIN1 interferes with cytokine-activated signaling pathways in experimental in vitro and ex vivo models of psoriasis

**Fig. 3:**
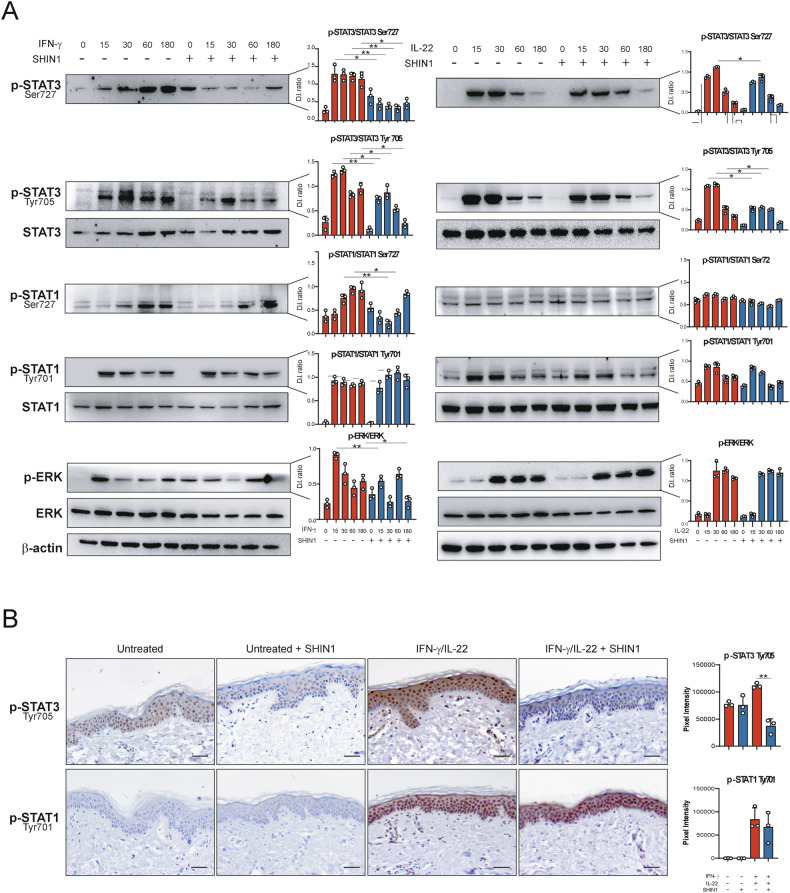
SHIN1 inhibits pro-inflammatory signaling pathways mediated by IFN-γ and IL-22 in keratinocyte cultures and in ex vivo skin grafts. **A** Western blotting analysis was performed on keratinocytes pre-treated with SHIN1 and subsequently stimulated with IFN-γ and IL-22 for the indicated time-points. Expression levels of p-STAT3 (Ser727; Tyr705), STAT3, p-STAT1 (Ser727; Tyr701), STAT1, p-ERK, ERK, and β-actin are shown. Graphs show the densitometric intensity (D.I.) values used to assess protein expression, with ratios of p-STAT1/total STAT1, p-STAT3/STAT3, and *p*-ERK1/2/ERK1/2, all normalized to β-actin expression. Data are presented as mean values ± SD from three independent experiments. *p < 0.05 and **p < 0.01. **B** IHC analyses for *p*-STAT1 Tyr701 and p-STAT3 Tyr705 (red/brown staining) were performed on paraffin-embedded sections of ex vivo skin grafts (*n* = 3) obtained from healthy skin and treated with IFN-γ/IL-22, with or without SHIN1. Sections were counterstained with Mayer’s Hematoxylin (H/E). Representative stainings are shown. Scale bars: 40 μm. Graphs show the mean pixel intensity values ± SD for each staining. Analyses were performed in four different fields per section. *p < 0.05 and **p < 0.01.

Proliferative and inflammatory events in psoriatic keratinocytes are regulated primarily by the transcription factors signal transducer and activator of transcription (STAT)3 and STAT1, which are activated by IL-22 and IFN-γ, respectively [26]. To determine whether SHMT inhibition affects these pathways, we analyzed STAT3 and STAT1 phosphorylation in cytokine-treated keratinocytes with or without SHIN1. Critical step in STAT3 and STAT1 activation is their phosphorylation on specific tyrosine residues (Tyr705 for STAT3 and Tyr701 for STAT1) and serine residues (Ser727 for both STAT3 and STAT1) [26]. We found that SHIN1 reduced the IFN-γ-induced phosphorylation of STAT3 at Ser727 and Tyr705 without affecting total amount of STAT3 protein (Fig. 3A, left panels). SHIN1 also modestly inhibited IL-22-induced phosphorylation of STAT3 at both Ser727 and Tyr705 residues (Fig. 3A, right panels). In parallel, SHIN1 slightly reduced IFN-γ-induced STAT1 Ser727 phosphorylation without affecting Tyr701 phosphorylation (Fig. 3A, left panels). Similarly, SHIN1 had only minimal effects on IL-22-induced STAT1 Tyr701 phosphorylation and did not modulate STAT1 Ser727 phosphorylation (Fig. 3A, right panels). Finally, SHIN1 exerted limited effect on extracellular signal-regulated kinase (ERK)1/2 signaling, a pathway involved in cell survival and self-protection from inflammation, activated by both IL-22 and IFN-γ in human keratinocytes (Fig. 3A). All the full-length blots of Fig. 3A are shown in the Supplemental files.

We next examined the effects of SHIN1 on molecular signaling pathways in the ex vivo psoriasis model. In particular, we examined the expression of Tyr705-phosphorylated STAT3 and Tyr701-phosphorylated STAT1 by immunohistochemistry (IHC) in skin explants activated with IL-22 and IFN-γ for 5 h, with or without SHIN1. As shown in Fig. 3B, IL-22 and IFN-γ markedly increased nuclear phosphorylated STAT3 and STAT1 in epidermal keratinocytes compared to untreated samples. Consistent with the in vitro data, SHIN1 significantly reduced phosphorylated STAT3, decreasing the number of STAT3-positive nuclei by approximately 2.4 folds, whereas phosphorylated STAT1 levels remained unchanged (Fig. 3B).

Taken together, these results indicate that SHIN1 preferentially attenuates STAT3 signaling in cytokine-activated human skin.

### SHIN1 restores proper proliferation and differentiation programs and reduces inflammatory gene expression in psoriatic skin explants

To validate the inhibitory effects of SHIN1 in human disease tissue, lesional skin biopsies from patients with plaque-type psoriasis (*n*=5) were cultured ex vivo with or without SHIN1 for 24 h.

A panel of pro-inflammatory genes expressed in psoriatic skin, along with proliferation and differentiation markers of keratinocytes, were analyzed by real-time quantitative PCR (RT-qPCR) and IHC, respectively.

**Fig. 4:**
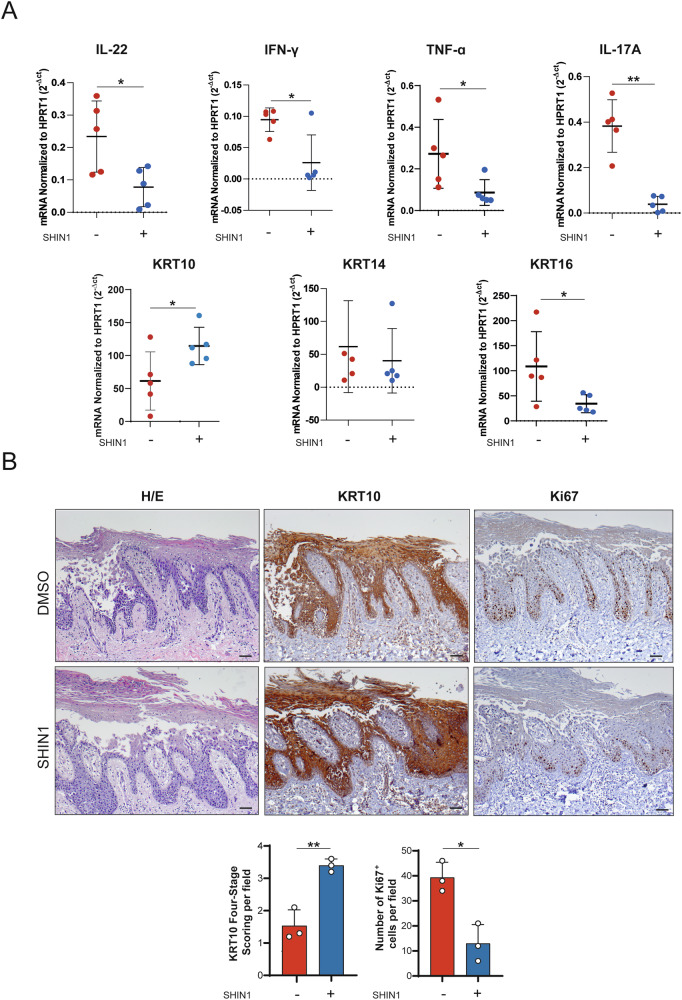
SHIN1 treatment of psoriatic skin explants restores proper proliferation and differentiation while reducing pro-inflammatory gene expression. **A** mRNA expression levels of IL-22, IFN-γ, TNF-α, IL-17A, KRT10, KRT14 and KRT16 mRNA expression levels were analyzed in skin biopsies obtained from lesional skin of psoriatic patients and cultured for 24 h, with or without SHIN1. *p < 0.05 and **p < 0.01. **B** Immunohistochemistry analyses for KRT10 and Ki67 (red/brown staining) were performed on paraffin-embedded sections of skin biopsies obtained from psoriatic lesional skin and treated or not with SHIN1 for 24 h. Sections were counterstained with Mayer’s Hematoxylin (H/E). Representative stainings are shown. Scale bars: 40 μm. Graphs show the mean values ± SD of four-stage score values for KRT10-positive and Ki67-positive cells, calculated from three sections for each patient. *p < 0.05 and **p < 0.01.

As shown in Fig. 4A, SHMT1/SHMT2 inhibition significantly reduced mRNA levels of IL-22, IFN-γ, TNF-α and IL-17A, all cytokines abundantly produced by different leukocyte subpopulations infiltrating psoriatic skin. The pro-inflammatory KRT16 was also reduced after SHIN1 treatment. Consistent with in vitro data, SHIN1 decreased epidermal proliferation, as indicated by reduced Ki67 immunoreactivity in basal keratinocytes, and restored expression of the differentiation marker KRT10 in suprabasal layers (Fig. 4B). SHIN1 exerted only modest and not significant effects on CXCL9, CXCL10, CCL2, CCL5, and IL-6 mRNA expression (Supplementary Fig. S1), possibly reflecting the complex inflammatory milieu within psoriatic plaques.

Together, these data demonstrate that SHIN1 promotes normalization of keratinocyte proliferation and differentiation while attenuating inflammatory activation in psoriatic skin.

## Discussion

Epidermal keratinocytes in psoriatic skin overproduce cytokines and growth factors that promote sustained activation of STAT3 and STAT1 signaling pathways [27, 28]. Among them, IL-22 and IFN-γ potently trigger STAT3 and STAT1 phosphorylation and have a pathogenetic role by altering keratinocyte proliferation, differentiation, and inflammatory responses [29]. IL-22 primarily acts on keratinocytes by promoting hyperproliferation and de-differentiation and by inducing the release of chemokines (CXCL1, CXCL2, CXCL8, CCL20), and AMPs (HBD-2, HBD-3, S100 proteins) [30, 31]. These effects are mediated mainly through STAT3, which is critically involved in epidermal alterations observed in both human psoriasis and experimental mouse models [27]. Although studies have demonstrated a central role for IL-22 in psoriasis pathogenesis, IL-22 regulates a more limited pro-inflammatory gene set than IFN-γ. Transcriptional profiling studies have in fact revealed a dominant IFN-γ signature in lesional psoriatic skin, with widespread activation of STAT1-dependent pathways [32]. Coherently, IFN-γ is the most potent inducer of keratinocyte inflammatory activation, regulating ~1,200 genes [33], including chemokines that recruit Th1/Th17 cells (CCL2, CCL5, CXCR3 ligands), DCs (CCL13, CCL20), and CCR10⁺ skin-homing T cells [34]. Moreover, IFN-γ upregulates ICAM-1, HLA-DR and MHC-I on keratinocytes, thereby facilitating lymphocyte retention and activation within psoriatic lesions [35, 36].

Despite its strong anti-proliferative activity, IFN-γ is paradoxically associated with epidermal hyperplasia and acanthosis [33]. This apparent discrepancy likely reflects the complex interplay between cytokines, particularly IL-22, which locally regulate the proliferative and differentiative processes in psoriatic keratinocytes. IFN-γ may also indirectly support keratinocyte proliferation by inducing IL-6 and, to a lesser extent, CXCL8, thereby partially compensating for its anti-proliferative action [37]. Together with inflammatory gene dysregulation, impaired responsiveness to IFN-γ has been attributed to a pathogenic imbalance between STAT1 and STAT3 signaling pathways [32].

In a previous study, we demonstrated that SHMT1- and SHMT2-dependent alterations in serine/glycine and folate metabolism exacerbate keratinocyte hyperproliferation and inflammation in vivo. Specifically, SHIN1, a folate-competitive SHMT1/2 inhibitor [22], impairs keratinocyte growth through purine depletion and reduces epidermal hyperproliferation and inflammatory gene expression in the IMQ-induced psoriasiform dermatitis [15]. SHIN1 blocks serine-derived one-carbon transfer into the THF cycle, thereby limiting the availability of one-carbon units required for nucleotide biosynthesis and reducing purine pools. Interestingly, methotrexate (MTX), a long-standing systemic therapy for psoriasis [38], acts through a similar folate-dependent mechanism. MTX inhibits dihydrofolate reductase, preventing folate regeneration and suppressing DNA/RNA synthesis [39]. Thus, SHIN1 treatment offers a proof-of-concept that selective inhibition of SHMTs can not only reproduce but also improve the therapeutic benefits of MTX in psoriasis while minimizing dose-related toxicity.

The inhibitory effect of SHIN1 appear to be mediated primarily by the SHMT2 isoform, which is selectively upregulated in psoriatic lesions, unlike SHMT1. Notably, SHMT2 upregulation appeared specific to psoriasis, as it is not increased in atopic dermatitis or basal cell carcinoma [15]. This specificity indicates that targeting SHMT-2-dependent pathways may represent a disease-selective therapeutic strategy. In the present study, we therefore investigated whether the inhibition by SHIN1 of SHMT-dependent metabolic pathways intersecting with IL-22 and IFN-γ can modulate proliferation, differentiation, immune molecule expression and cytokine-dependent pathogenic pathways in experimental models of psoriasis. The study was conducted using in vitro and ex vivo models of skin inflammation that mimic psoriasis, based on keratinocyte cultures and skin explants activated with IL-22 and IFN-γ. Of note, SHIN1 inhibitor was also used to treat skin biopsies from psoriasis plaques of patients. The latter ex vivo model is extremely valuable and unique for studying human psoriasis, allowing direct investigation of underlying mechanisms and potential therapeutic interventions.

Consistent with our previous findings [15], SHIN1 markedly reduced basal keratinocyte proliferation and S-phase entry and exerted an even stronger inhibitory effect under IL-22 stimulation. SHIN1 profoundly impaired IL-22-induced keratinocyte growth, likely by limiting the availability of metabolic intermediates required for protein and nucleotide synthesis and by inducing the cell-cycle inhibitors p21 and p27 [15]. SHIN1 also potentiated the cytostatic effects of IFN-γ, which are largely mediated by p21, further supporting the role of serine metabolism in regulating keratinocyte cell-cycle progression. Importantly, these anti-proliferative effects were confirmed not only in vitro but also in psoriatic skin explants, where SHIN1 reduced the number of Ki67-positive basal keratinocytes.

In parallel, SHIN1 was able to restore keratinocyte differentiation in ex vivo psoriatic skin explants, as evidenced by the recovery of KRT10 expression in suprabasal layers. These findings extend our previous observations in the IMQ-induced psoriasis mouse model, in which topical SHIN1 re-established epidermal differentiation [15]. In contrast, SHIN1 did not restore differentiation in IL-22-treated keratinocytes in vitro, likely reflecting the severe growth impairment induced by SHMT inhibition under these conditions.

Whether SHIN1 acts directly on keratinocytes or indirectly by modulating T cells that release cytokines controlling proliferation and differentiation remains to be clarified. Indeed, SHIN1 may exert indirect effects by limiting the expansion of IL-22-producing T cells, thus preventing IL-22 local release. Consistently, serine has been reported as an essential metabolite for T cell proliferation [17]. A previous paper showed that antigen-specific T cell expansion is reduced when serine availability is limited [17]. These findings were confirmed in vivo, where antigen-specific T cell proliferation was compromised after restriction of dietary serine [17]. In the future, it will be crucial to test SHIN1 effect on T lymphocytes, providing additional insights into its potential therapeutic effects.

In this study, we also investigated the impact of modulating serine metabolism on IFN-γ-driven inflammatory responses in keratinocytes and found that SHIN1 efficiently inhibited this pathogenic pathway, reducing the expression of CXCL9, CXCL10, CCL2, CCL5, IL-6, and HLA-DR. In contrast, SHIN1 had only modest or null effects on inflammatory molecules mainly induced by IL-22, such as CXCL8, HBD-2 and S100A7. These results were confirmed in both in vitro and ex vivo conditions, specifically in cytokine-activated keratinocyte cultures and skin explants. The effect of SHIN1 was selective since not all the genes analyzed were significantly influenced by treatments. The genes most sensitive to SHIN1 action were those transcriptionally co-regulated by STAT3 and STAT1 (CXCL10, CXCL9, CCL2, CCL5, IL-6), whereas genes mainly controlled by STAT1 or STAT3 alone, or in combination with IRF-1, NF-κB, or AP-1 (CXCL8, HBD-2, S100A7) [40–43] were less influenced. Consistently, SHIN1 mainly impaired STAT3 full activation in keratinocyte cultures following IL-22 and IFN-γ treatments, as well as STAT1 Ser727 phosphorylation induced by IFN-γ. Importantly, STAT3 inhibition was also confirmed in ex vivo skin explants activated with IL-22 and IFN-γ.

Mechanistically, SHIN1 effects on STAT3 may depend not only on serine levels but also on redox balance and glutathione (GSH) synthesis, as SHMT2 inhibition markedly reduces GSH levels [15]. Since STAT3 phosphorylation relies on oxidative stress and S-glutathionylation of cysteine residues [44], SHIN1 could regulate STAT3 activation through the regulation of GSH levels. Consistent with this hypothesis, our previous studies showed that dehydrocostuslactone (DCE) and costunolide (CS), two terpenes naturally occurring in many plants, sequestered and reduced intracellular GSH in human keratinocytes and decreased STAT3 full activation in response to IL-22 or IFN-γ [45, 46]. Other molecules influencing GSH levels, such as 1-buthionine sulphoximine (BSO), also regulate STAT3 and ERK1/2, although they decrease synthesis of GSH rather than sequestering and oxidizing it. Interestingly, GSH modulation can have opposite effects depending on cell type and stimuli. For instance, BSO inhibits STAT3 or ERK1/2 induced by leukemia inhibitory factor in cardiac myocytes, whereas it has weak effects on IL-6-induced STAT3 in endothelial cells [47, 48].

In this study, we also found that STAT3 inhibition by SHIN1 was accompanied by decreased STAT1 phosphorylation in Ser727 and ERK1/2 phosphorylation. However, STAT1 and ERK1/2 were only slightly downregulated, suggesting that SHIN1 effect could be indirect. Indeed, other GSH modulators such as DCE, CS and BSO can inhibit STAT1 activation by regulating upstream JAK tyrosine kinases, rather than directly modulating STAT1 phosphorylation. DCE and CS also inhibit MAP kinases-related pathways, including ERK1/2 [45, 47, 49] and enhance the epidermal growth factor receptor phosphorylation, which triggers anti-inflammatory and anti-apoptotic responses in IFN-γ/TNF-α-treated keratinocytes [50]. Our data demonstrates that the principal effect of SHIN1 on keratinocytes is the attenuation of STAT3 activation, consistent with its impact on downstream signaling. This inhibition may downregulate IL-22–mediated genes involved in proliferation, such as cyclin D1, PCNA and retinoblastoma protein, similar to effects of DCE and CS [46]. Consistently, we showed reduced Ki67 and the barrier alarmin KRT16 expression in psoriatic skin explants treated with SHIN1.

Beyond its effects on keratinocytes, SHIN1 also reduced the expression of key pathogenic cytokines, including IL-17A, IFN-γ, TNF-α and IL-22, in psoriatic skin explants, indicating a broader immunomodulatory activity. These findings suggest that modulation of serine metabolism may simultaneously restrain keratinocyte hyperproliferation and attenuate inflammatory cytokine production by infiltrating immune cells. Consistently, recent studies have linked serine metabolism to macrophage inflammatory polarization, demonstrating that inhibition of de novo serine synthesis reduces IL-1β production and inflammatory phenotypes [51].

In conclusion, our study identifies serine/glycine metabolism as a critical regulator of cytokine-driven keratinocyte hyperproliferation and inflammation in psoriasis. By selectively inhibiting SHMT enzymes, SHIN1 attenuates STAT3 and STAT1 signaling pathways associated with IFN-γ and IL-22, normalizes keratinocyte proliferation and differentiation, and suppresses pathogenic inflammatory programs. Our findings, together with previous studies identifying SHIN1 as a negative modulator of psoriasiform reactions in IMQ mouse model [15], provide a strong rationale for the development of SHIN1-based topical formulations and other metabolism-targeted therapeutic strategies for psoriasis and potentially for other IL-22-driven hyperproliferative skin disorders, including NMSC [52]. In this context, targeting serine metabolism could restrain aberrant keratinocyte growth induced by infiltrating immune cells, rendering the epidermis less susceptible to cytokine-induced damage.

## Materials and methods

### Keratinocyte cultures and treatments

Human keratinocyte cultures were obtained from healthy subjects undergoing plastic surgery (n = 3; two females and one male, aged 40–60 years), after patients’ informed written consent and with the approval of the IDI-IRCCS Local Ethics Committee (Prot. 581-2/2019, approved on July 15, 2019), in accordance with the Declaration of Helsinki Guidelines. Specifically, cells were enzymatically isolated from skin and then were seeded (1.2–2 × 10^4^/cm^2^) on a feeder layer of irradiated 3T3 fibroblasts and cultured as previously reported [25, 46]. Otherwise, neonatal human epidermal keratinocytes (HEKn), purchased from Invitrogen-Thermo Fisher (Carlsbad, CA, USA), were also used for the present study.

Second-passage keratinocyte cultures were grown in Keratinocyte Growth Medium (KGM, Clonetics, San Diego, CA, USA) for at least 3–5 days (about 70% confluence) and then, serum-starved for 18 h in Keratinocyte Basal Medium (KBM), prior to treatments. Before cytokine stimulation, cultures were pre-treated for 1 h with 25 μM 6-amino-1,4-dihydro-4-[5-(hydroxymethyl) (SHIN1), a serine hydroxymethyltransferase 1 and 2 (SHMT1/SHMT2) inhibitor (MedChemExpress, Monmouth Junction, NJ, USA), IUPAC name:

6-amino-1,4-dihydro-4-[5-(hydroxymethyl)[1,1'-biphenyl]-3-yl]-3-methyl-4-(1-methylethyl)pyrano[2,3-c]pyrazole-5-carbonitrile) [22]. Keratinocyte cultures were stimulated with recombinant human (rh) IL-22 (70 ng/ml), IFN-γ (200 U/ml) and IL-17A (50 ng/ml), all purchased from R&D Systems (Minneapolis, MN, USA), at different time points, either alone or in combination, depending on the experimental setup.

To induce terminal differentiation of keratinocytes, cells were plated at 5000/cm² in 6-well plates, grown in KBM until reaching 100% confluence (t0), and then maintained in culture for an additional four days post-confluence [53]. High-confluence cultures were stimulated with rh 200 U/ml IFN-γ or 70 ng/ml IL-22 in KBM, with or without 25 μM SHIN1, which was administered by pre-treating the cultures for 1 h prior to cytokine stimulation.

### Ex vivo skin models and biopsies

Ex vivo skin models were established by using 6-mm punch skin explants of healthy volunteers (*n* = 3) undergoing plastic surgery at IDI-IRCCS (Prot. 581-2/2019, approved on July 15, 2019). Otherwise, 8-mm punch biopsies were taken from lesional skin of patients affected by plaque-type psoriasis (PASI score from 10 to 40) (*n* = 5), with approval from the IDI-IRCCS Local Ethics Committee (Prot. 727-1/CE/2023, approved on March 15, 2023). All participants gave their informed written consent.

Explants from healthy skin were treated for 5 or 18 h with a combination of IL-22 (250 ng/ml) and IFN-γ (1000 U/ml), in presence of 50 μM SHIN1 or vehicle alone. Samples were divided into two equal parts for subsequent IHC and RT-qPCR analyses.

Skin specimens from psoriatic skin were divided into four equal parts, which were cultured for 24 h in the presence of 50 µM SHIN1 (two parts) or vehicle alone (two parts) in RPMI complete medium supplemented with 10% fetal bovine serum. The samples were then processed for subsequent IHC and RT-qPCR analyses.

### Proliferation assays

Keratinocyte cultures isolated from healthy subjects were seeded (1 × 10^4^ cells/well) into 96-well cell plates and stimulated for 24, 48 and 72 h with IFN-γ or IL-22 cytokines, in presence or not of 25 μM SHIN1. Proliferation was evaluated by using CyQuant cell proliferation kit (Thermo Fisher Scientific, Waltham, MC, USA), which assesses total DNA content by measuring fluorescence emission at 530 nm in the EnSight multimode plate reader (Perkin Elmer, Waltham, MC, USA).

Additionally, proliferation of HEKn was evaluated by pulse-labeling cultures grown in KGM for 3 h with 10 μM 5-bromo-2'-deoxyuridine (BrdU) analogue EdU, which is incorporated into neo-synthetized DNA of dividing cells. IFN-γ or IL-22 cytokines were administered with or without the SHIN1 inhibitor for 24 h and then processed using the Click-iT EdU Alexa Fluor 488 Flow Cytometry Assay Kit (Invitrogen, Thermo Fisher) for flow cytometry analysis conducted on a CytoFLEX cytometer (Beckman Colter, Brea, CA, USA).

### RNA isolation and RT-qPCR analysis

Inflammatory gene expression analysis was performed using total RNA extracted from cultured keratinocytes treated with SHIN1 and 18-h stimulated with IL-22, IFN-γ and IL-17 cytokines, as well as from both healthy and psoriasis skin explants. TRIzol reagent (Invitrogen-Thermo Fisher) and RNeasy Lipid tissue kit (Qiagen, Chatsworth, CA, USA) were used to extract RNA from cultured cells and skin explants, respectively. mRNA was reverse transcribed into cDNA using iScript cDNA synthesis kit (Bio-Rad, Hercules, CA, USA) and analyzed by qPCR. The expression of inflammatory genes and keratins was evaluated in the ABI PRISM SDS 7000 PCR Instrument (Applied Biosystems, Branchburg, NJ, USA), using SYBR Green or Taqman PCR reagents. The primer pairs used in qPCR reactions were as follows: for CXCL10, Fw 5’- GG CAT TCA AGG AGT ACC TCT CT -3’ and Rev 5’-CTG ATG CAG GTA CAG CGT ACG -3’, for CXCL9, Fw 5’-TCA CAT CTG CTG AAT CTG GG-3’ and Rev 5’-CCT TAA ACA ATT TGC CCC AA-3’, for CCL5, Fw 5’- CTA CTG CCC TCT GCG CTC C -3’ and Rev 5’- TGG TGT CCG AGG AAT ATG GGC-3’, for CCL2, Fw 5’-CAC CAG CAG CAA GTG TCCC- 3’ and Rev 5’-CCA TGG AAT CCT GAA CCC AC- 3’, for IL-6, Fw 5’-GGC ACT GGC AGA AAA CAA CC-3’ and Rev 5’-CAC CAG GCA AGT CTC CTC AT-3’, for CXCL8, Fw 5’- CTC TGT GTG AAG GTG CAG TTT T -3’ and Rev 5’-GGG TGG AAA GGT TTG GAG TAT G -3’, for HBD-2, Fw 5’ - TCC TCT TCT CGT TCC TCT TCA TAT T - 3’, Rev 5’- TTA AGG CAG GTA ACA GGA TCG C- - 3' and 5’-ACC ACC AAA AAC ACC TGG AAG AGG CA-3’ as internal probe, for S100A7, Fw 5’- AGAAGCCAAGCCTGCTGACGAT-3’ and Rev 5’- GTCCTTTTTCTCAAAGACATCGGC -3’, for IL-22, Fw 5’ - GCA GGC TTG ACA AGT CCA ACT- 3' and Rev 5’- GCC TCC TTA GCC AGC ATG AA- 3', for TNF-α, Fw 5’- CTC TTC TGC CTG CTG CAC TTT G- 3' and Rev 5’- ATG GGC TAC AGG CTT GTC ACT C-3’, for IL-17A, Fw 5’- GGA CTG TGA TGG TCA ACC TGA- 3' and Rev 5’-TCA TGT GGT AGT CCA CGT TCC- 3', for KRT10, Fw 5’- TCC CAA CTG GCC TTG AAA CA- 3' and Rev 5’- TGA GAG CTG CAC ACA GTA GC- 3', for KRT16, Fw 5’- GAG ATC AAA GAC TAC AGTCC- 3' and Rev 5’- CAT GCT CAT ACT TGG TCC TG- 3'. Primers for HPRT-1 mRNA, 18S rRNA, KRT14 and IFN-γ were provided by Applied Biosystems (HS4333768 and HS99999901_s1, HS00559328_m1, HS00174143_m1 respectively). The levels of gene expression were determined by normalizing HPRT1 and 18S rRNA expression. The values obtained from triplicate experiments were averaged and reported as mean 2^-ΔCT ± SD relative to untreated controls, which were arbitrarily assigned a value of 1.

### Western blotting analysis

Western blotting was performed on protein extracts obtained from keratinocytes 1-h pre-treated with 25 μM SHIN1 and then stimulated with IFN-γ or IL-22 at different time-points. Total proteins were extracted by solubilizing cells in RIPA buffer and quantified using the Protein Assay Dye Reagent (Bio-Rad). Equal amounts of total protein (30 µg per lane) were resolved by SDS-PAGE, and consistent loading was maintained for all samples and gels. Western blotting filters were developed using the ECL-plus detection system (Santa Cruz) or the SuperSignal West Femto kit (Pierce, Rockford, IL). The Abs employed for the study were as follows: anti-KRT10 and anti-loricrin from Covance (Emeryville, CA, USA); anti- p-STAT1 (Ser727, #9177), anti-p-STAT1 (Tyr701, #7649), anti-p-STAT3 (Tyr705, #9131), all provided by Cell Signaling Technology; anti-ΔNp63 (#sc8431), anti-p-STAT3 (Ser727, #sc-135649), anti-STAT1 (#sc-346), anti-STAT3 (sc-482), anti-ERK1/2 phosphorylated in Tyr 204 (#sc-7383), ERK1/2 (#sc-93) and anti-β-actin (#sc-1615) all purchased from Santa Cruz (Dallas, TX, USA). Filters were developed with anti-mouse, anti-goat, or anti-rabbit Ig Abs conjugated to HRP, depending on the primary Abs. Immunoblots were analyzed by using the ChemiDoc MP Imaging System (Bio-Rad) and quantifications of protein bands was performed by densitometric analysis by using the Image J software (version 1.54f, https://imagej.nih.gov/ij/). Bands intensities were evaluated in three independent experiments and reported as means of densitometric intensity ± SD.

### Flow cytometry analysis

Keratinocytes were analyzed for ICAM-1, HLA-DR and major histocompatibility complex (MHC) class I membrane expression by flow cytometry. Cultured cells were pre-treated with 25 μM SHIN1 and then stimulated for 24 h with IFN-γ, IL-22, and/or IL-17. Membrane protein expression levels were evaluated using APC-conjugated anti-CD54/ICAM-1 (clone 84H10; Immunotech, Marseille, France), anti-HLA-DR (clone L243, BD Pharmingen, Franklin Lakes, NJ, USA) and anti-human MHC class I (clone 51-10C9, BD Pharmingen) monoclonal Abs. Cells were analyzed by the Accuri C6 Flow cytometer (BD Pharmingen) equipped with Cell Quest software (BD Pharmingen).

### Immunohistochemistry

Human skin samples were fixed with 10% formalin, prior to embedding in paraffin. 5-μm sections were dewaxed and rehydrated, then incubated with primary anti-p-STAT1 (Tyr701, #9167, Cell Signalling Technologies), anti-p-STAT3 (Tyr705, #9145, Cell Signalling Technologies), anti-KRT10 from Covance (Emeryville, CA, USA), anti-Ki67 (#M724001-2, Dako, Glostruk, Denmark) Abs. Secondary biotinylated Ab and staining kits (Vector Laboratories, Burlingame, CA, USA) were used to develop immunoreactivities. Sections were counterstained with Mayer’s H&E and were visually analysed by two pathologists experienced in dermatology, and positivity was evaluated. For each skin specimen, two sections were analyzed, and positive cells were counted in four adjacent fields at a magnification of 200X. Immunohistochemistry images were acquired using Axioplan 2 microscope with Axiocam 503 color microscope camera (Zeiss, Oberkochen, Germany) and processed with ZEN 2 lite imaging software (Zeiss). A quantitative analysis of staining was performed by using the Image J software (version 1.54f; https://imagej.nih.gov/ij/).

### Statistical analysis

Normality distribution and homogeneity of variances between the experimental groups were assessed prior to statistical analysis. Datasets that met these assumptions were analyzed using parametric tests. Specifically, for in vitro and ex vivo studies, one-way ANOVA was used for proliferation assays, as well as for mRNA and protein expression analyses. *Post-hoc* multiple comparisons were performed using Tukey’s test for ANOVA analyses. Data of skin explants were analyzed using the Student’s t-test. All analyses were conducted using GraphPad Prism v.8.0 (GraphPad Software, La Jolla, CA, USA). Data are presented as mean ± SD, and statistical significance was considered at *p* ≤ 0.05.

## Supplementary information

Supplementary Figure S1

Original Western Blots of Fig.1B

Original Western Blots of Fig.3A

## Data Availability

The datasets generated and analysed during the current study are available from the corresponding author (c.albanesi@idi.it) on reasonable request.
