# Supplementary figures and images for: Targeting serine/glycine metabolism to attenuate IFN-γ- and IL-22-driven inflammation and hyperproliferation in psoriasis
Source: Cell Death Discov. 2026 May 8;12:289. doi: 10.1038/s41420-026-03138-3 (PMC13323995; doi:10.1038/s41420-026-03138-3)

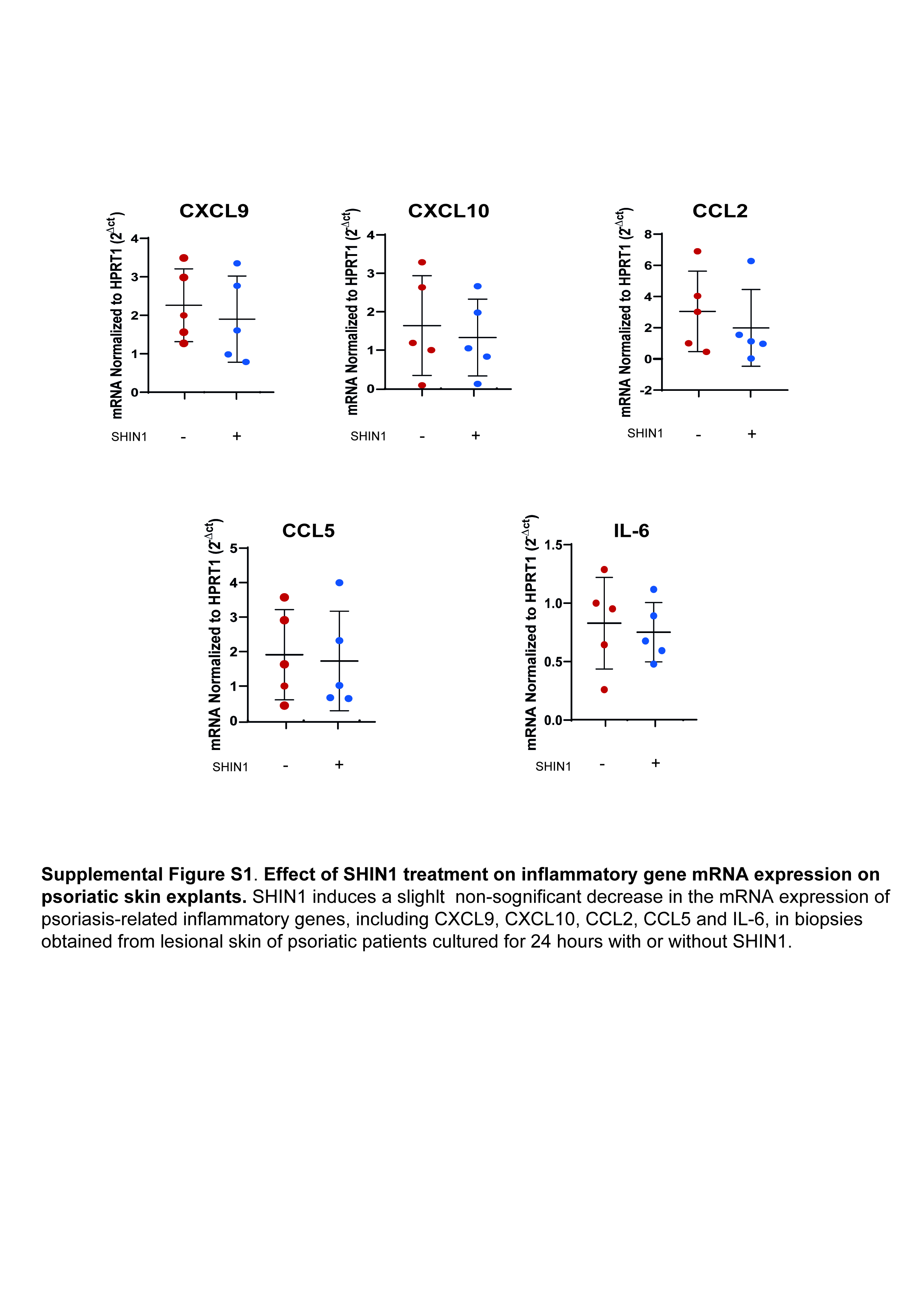

Supplement: Supplementary file 1 — Supplementary Figure S1 [file 41420_2026_3138_MOESM1_ESM.tif]

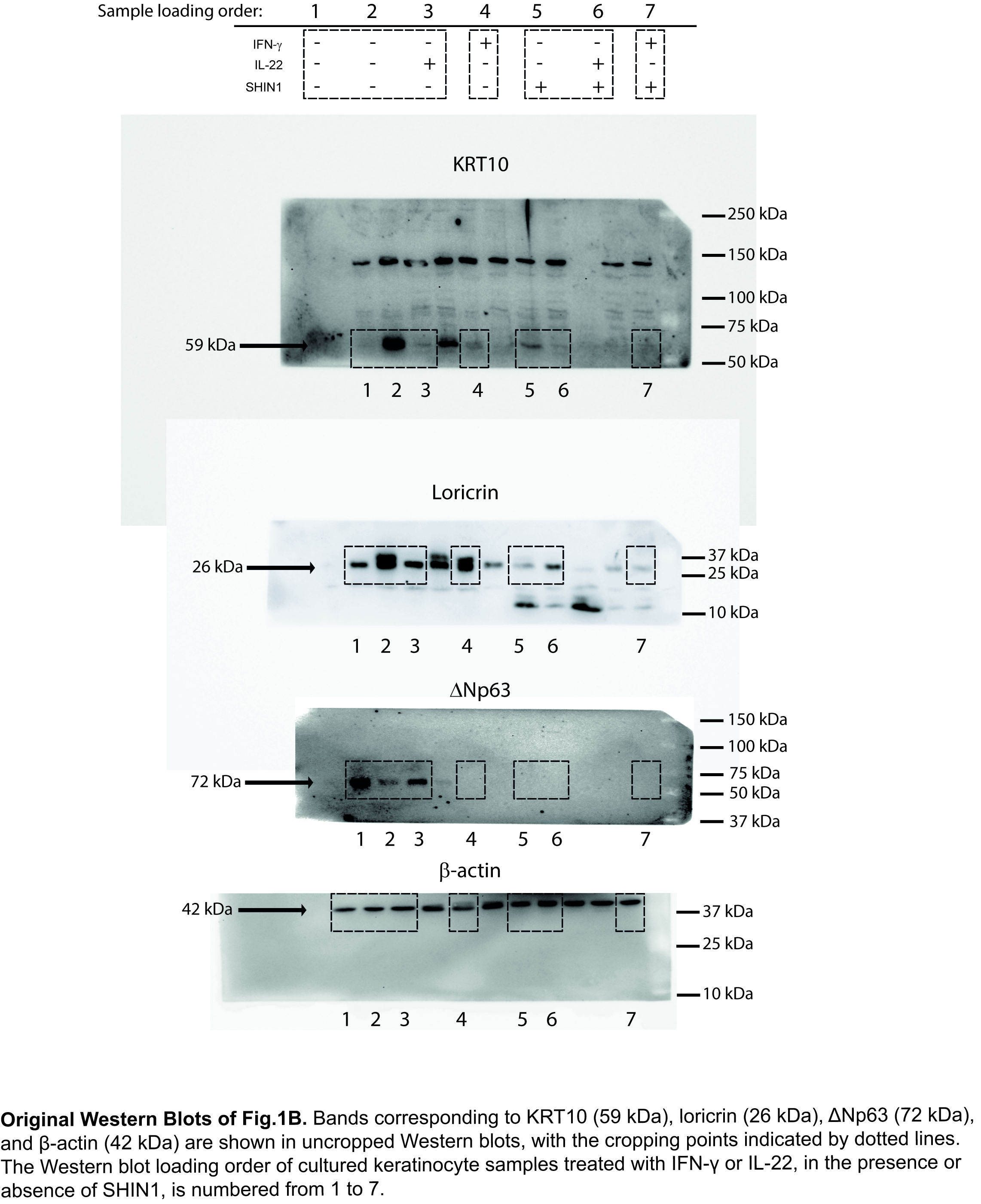

Supplement: Supplementary file 2 — Original Western Blots of Fig.1B [file 41420_2026_3138_MOESM2_ESM.tif]

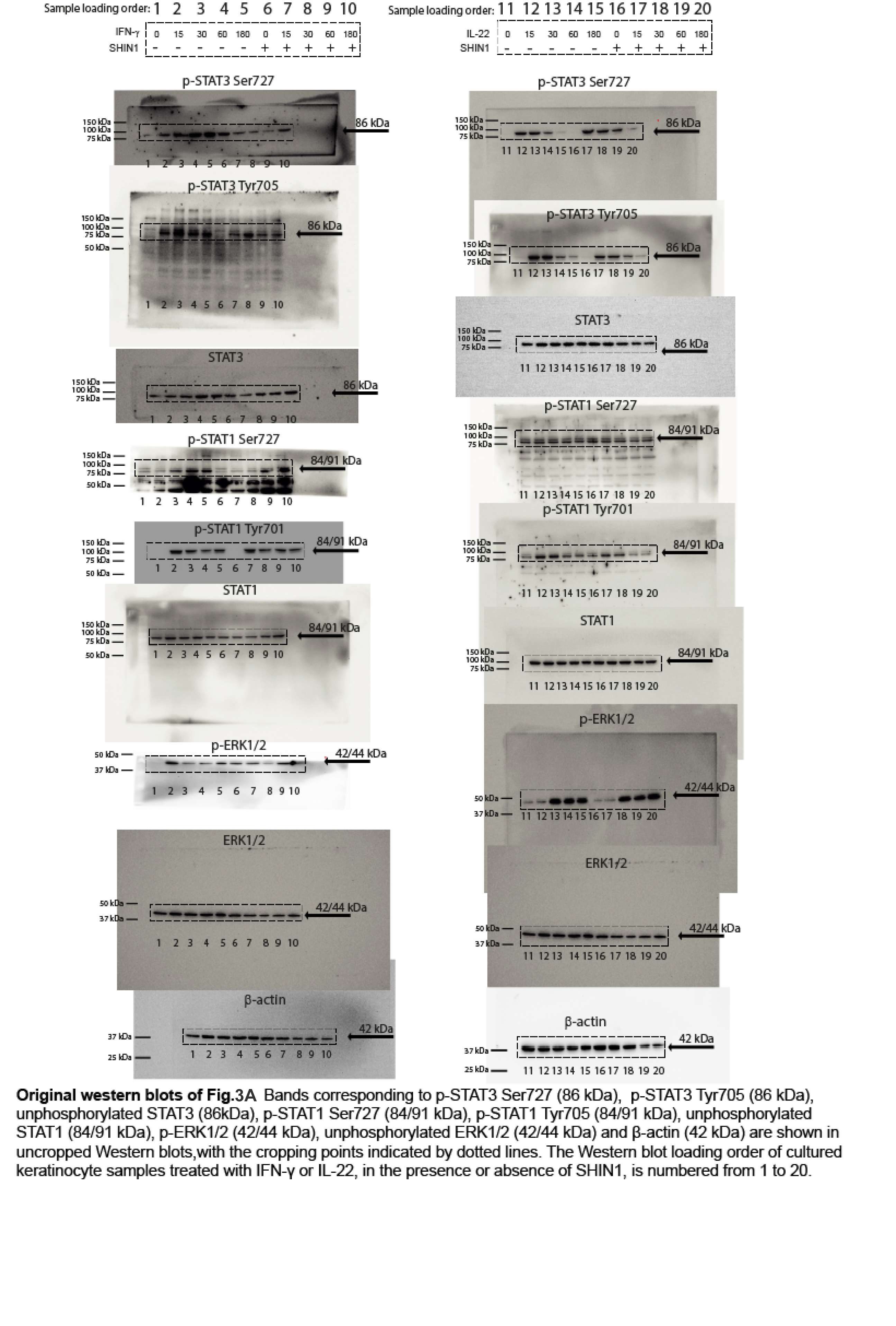

Supplement: Supplementary file 3 — Original Western Blots of Fig.3A [file 41420_2026_3138_MOESM3_ESM.tif]
